# UV Monitoring for Public Health

**DOI:** 10.3390/ijerph15081723

**Published:** 2018-08-11

**Authors:** Mario Blumthaler

**Affiliations:** Medical University Innsbruck, Division for Biomedical Physics, 6020 Innsbruck, Austria; mario.blumthaler@i-med.ac.at; Tel.: +43-512-9003-70871

**Keywords:** UV measurements, UV Index, web presentation

## Abstract

Overexposure to solar ultraviolet (UV) radiation is a risk for public health. Therefore, it is important to provide information to the public about the level of solar UV. The UV-Index (UVI) is the relevant quantity, expressing the erythemally weighted irradiance to a horizontal plane on a simple scale. As solar UV irradiance is strongly variable in time and space, measurements within a network provide the best source of information, provided they can be made available rapidly. However, to ensure the information is reliable, strict quality assurance/quality control (QA/QC) procedures for the monitoring networks are necessary. Near real time presentation of the measured UVI on web-pages is the best way to inform the public. The interpretation of the data in terms of the individual ‘allowable’ exposure time is heavily impacted by skin type, behavior, and clothing, and must be learned for each person through experience and guidance. Nonetheless, reliable knowledge of the actual level of the intensity of erythemally weighted irradiance and its variability forms the basis of education and public awareness. The challenges and requirements in providing comprehensive UVI data for public health guidance are here considered.

## 1. Introduction

**Figure 1:**
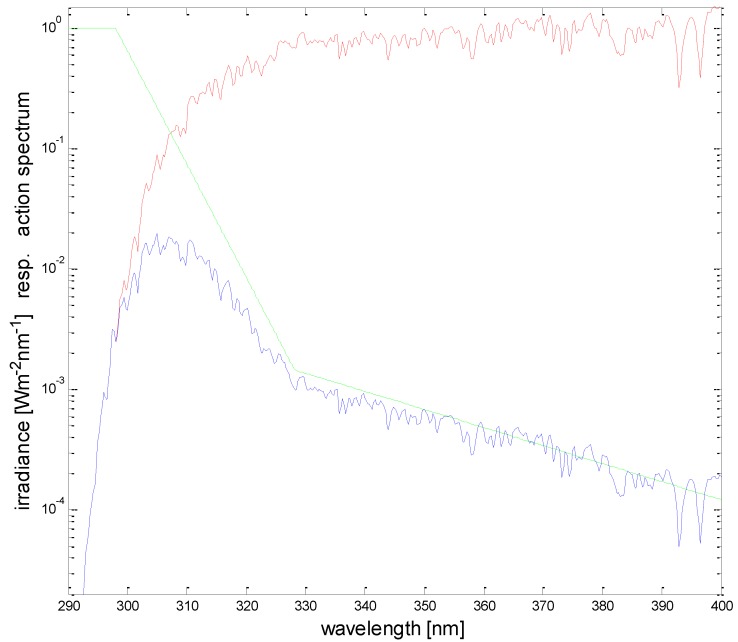
Erythema action spectrum (**green**), typical solar spectrum (**red**), and erythemally weighted solar spectrum (**blue**). The erythemally weighted irradiance is the integral under the blue curve.

The overexposure to solar UV is a risk for public health. Typical detrimental effects are the acute response of erythema (sun burning) and the longer-term risk of skin cancer. In addition, effects of sun related eye diseases show an acute response of snow blindness and the long-term risk of cataract formation. For assessing the exposure dose, two factors are decisive: intensity of the solar UV radiation (irradiance) and duration of the exposure. The product of these two factors determines the dose. Consequently, for a given dose under high irradiance, the exposure time has to be shorter than under lower irradiance. The absolute quantity of the dose that leads to overexposure is a very individual value. It depends on the skin type and on any pre-existing tanning of the skin. Therefore, a reasonable sub-erythemal dose limit is not a constant value even for the same person: usually in spring it is lower than in autumn due to build-up of natural protection (tanning, skin thickening) during the summer. Consequently, assessing the individual ‘allowed’ dose becomes a matter of personal preference and experience. However, it should not be overlooked that a small dose of UV-B exposure (wavelength range 280 nm to 315 nm) is essential for the formation of Vitamin D in human skin, but this dose is significantly lower than the dose to produce erythema. In order to control the exposure dose, one has to control the exposure time, but in addition, the irradiance has to be known. The irradiance is a physical quantity, which is measured with physical instruments. In order to use a quantity that is relevant for health applications, the physical level of irradiance is weighted with the biological action spectrum of erythema, which is generally taken as an indicator of detrimental health effects from UV radiation. The sensitivity of the human skin to erythema is strongly dependent on the wavelength of the irradiance, where shorter wavelengths in the UV-B wavelength range are more efficient in producing erythema than the longer wavelengths in the UV-A range (315 nm to 400 nm). Although this spectral dependency (‘erythema action spectrum’) is somewhat different for different persons, it is standardized by international organizations [1,2,3], so that the term ‘erythemally weighted’ irradiance is unique all over the world (Figure 1). In order to determine the erythemally weighted irradiance, the erythema action spectrum is multiplied at each wavelength with the absolute spectrum of the solar irradiance and then the resulting spectral curve is integrated over the wavelength range 280 nm to 400 nm. The unit of the erythemally weighted irradiance is communicated with the so-called ‘UV-Index’ (UVI), which by international definition is determined by scaling the physical unit (Wm^−2^) with a factor 40 [4]. The scaling factor is defined in such a way that the UVI reaches values between 8 and 10 at mid-latitudes in summer. Under special conditions, the UVI can be even higher than 20, e.g., in the South American Andes [5].

The classification and color coding of the UVI by the World Health Organization [4] is: 1, 2: low, green; 3, 4, 5: moderate, yellow; 6, 7: high, orange; 8, 9, 10: very high, red; 11+: extreme, violet. Only for low UVI is no protection suggested, otherwise increasing public heath campaigns to encourage protection includes seeking shade, slipping on a shirt, slopping on sunscreen, and slapping on a hat (the ‘slip, slop, slap’ campaign was launched in Australia in 1981 by Cancer Council Victoria [6]).

While the need for public health campaigns and the consequences of overexposure receive much publicity, the provision of accurate and reliable knowledge about the solar irradiance that underpins safety information given to the public is less well recognized. The efforts required for the comprehensive provision of UVI data are described below. Furthermore, it is intended to give a generic overview of the present situation of UV monitoring for public health, discussing the challenges and requirements and the available options for this purpose.

## 2. Variability of Solar UV Irradiance

Solar UV irradiance is highly variable in time and space. The most important controlling parameter under cloudless conditions is the elevation of the sun above the horizon. This is determined by the time during the day, the day within the year, and the geographic latitude. UV-B irradiance is more strongly dependent on solar elevation than visible radiation, because at low solar elevation the long path length of the rays through the atmosphere causes especially strong absorption of UV-B wavelengths by the ozone content in the atmosphere. As a consequence, e.g., the irradiance of erythemally weighted irradiance at mid-latitudes in winter is only about 1/18 of the summer value, whereas this ratio for visible radiation is much greater at 1/5 [7]. Thus, the levels of erythemally weighted irradiance in winter at mid-latitudes are almost negligible.

The second most important factor for erythemally weighted irradiance is the total amount of ozone in the atmosphere. This is variable with both time and location and can have variations of more than 20% within a few days. The relation between ozone variations and variations of the erythemally weighted UV irradiance is given by the ‘radiation amplification factor’: a decrease in total ozone by 1% will result in an increase in erythemally weighted irradiance by about 1.1% [8].

Further significant factors influencing erythemally weighted irradiance are the amount and type of aerosols and the albedo of the surroundings. Aerosols affect UV irradiance more than visible irradiance and can lead to reductions by 20 or 30% in heavily polluted regions [9]. The albedo of the surroundings will lead to an increase in irradiance in a reflective environment, most notably if the terrain is covered by snow. This increase in irradiance due to snow depends on the wavelength and is most pronounced at about 320 nm. A change in the terrain from snow free conditions to fresh snow covered conditions will increase the albedo at 320 nm by up to 90% and the erythemally weighted irradiance will increase by about 25% [9].

Finally, solar irradiance will increase with increasing altitude above sea level, and this increase is most pronounced in the UV-B wavelength range. This increase is caused by lower air density and thus by less attenuation due to scattering by air molecules (a process known as Rayleigh scattering that is proportional to the inverse fourth power of wavelength). Furthermore, at higher altitudes the amount of attenuating tropospheric ozone and aerosols is usually smaller and sometimes in addition the albedo is higher due to snow covered terrain. All these influencing factors are quite variable, and therefore, the range of the increase of erythemally weighted irradiance for an increase of altitude by 1000 m is between 10 and 20% [10]. In contrast, for visible irradiance, the increase is less than half of this value.

All the variations discussed so far are valid for cloud free conditions. If the sun is covered by clouds, irradiance at all wavelengths is reduced, and depending on the thickness of the clouds, this reduction can be down to 5 or 10% of the cloud free case. Thereby, UV irradiance is somewhat less attenuated than visible irradiance [11,12,13,14,15]. If the sun is not covered by clouds but clouds are nearby in the sky, then an enhancement of solar irradiance can be observed due to reflections of sunlight at the sides of the clouds. This enhancement is more pronounced at visible wavelengths than at UV wavelengths and during short periods it can reach up to 20% (e.g., [16,17]).

As a consequence of the strong variability of solar UV irradiance, it is difficult to calculate its level even for the cloudless case. Radiative transfer modelling (e.g., Reference [18]) can very accurately determine the irradiance, if all parameters describing the atmosphere and the surrounding are exactly known. However, because of incomplete knowledge of some parameters (especially aerosol characteristics, temperature profile up to the stratosphere, ground albedo distribution) uncertainties of up to 5–10% will result even in the best case [19]. It is almost impossible to accurately calculate actual levels of UV irradiance under cloud cover or under broken clouds. Estimation of erythemally weighted irradiance derived from a series of on-board satellite measurements (e.g., Reference [20]) also suffer from a lack of detailed knowledge of influencing parameters at specific locations, and furthermore, in most cases are limited to one daily overpass by the satellite. However, such retrievals have the great advantage of global coverage. Nonetheless, local measurements are the best way to get accurate information about the level of erythemally weighted irradiance to a horizontal plane and its diurnal and seasonal variation, and in addition allow for satellite product validation and ground-truthing.

## 3. Monitoring Requirements

Measurement of solar erythemally weighted irradiance is a difficult task, because the absolute energy in the UV-B wavelength range is very small, and the spectral energy at nearby longer wavelengths is higher by orders of magnitude. Therefore, high spectral purity of the measurement is necessary in order to avoid the disturbing influence of radiation at longer wavelengths (stray light).

The most accurate measurement of erythemally weighted irradiance is carried out with a high-resolution double monochromator spectroradiometer [21]. These very sophisticated instruments deliver full spectral information, which allows application of any desired spectral weighting function to calculate biologically relevant irradiance for e.g., erythema formation or vitamin D production. Common spectroradiometer are the Brewer Mark II and III (Kipp&Zonen, Delft, The Netherlands) or the DTM series (Bentham Instruments Limited, Reading, UK). The Brewer spectrometer is especially designed to operate in harsh environments, the procedures for calibration, data acquisition, and data analyses are well established. Typical data products are total ozone, NO_2_, aerosol optical depth, and spectral UV irradiance. Common double monochromator spectroradiometers are designed for indoor use and therefore they need specific adaptations for outdoor operation, e.g., special input optics connected with a quartz fiber to the monochromator and temperature stabilization of the mechanical and electronic parts of the instrument. Generally, spectroradiometer are expensive and need operation by experienced personnel. Furthermore, collecting a full spectrum in the UV often takes several minutes, during which the irradiance may change because of moving clouds. Therefore, spectroradiometers are not often used for monitoring purposes but for specific high quality measurements and as a reference instrument for less demanding measurements.

**Figure 2:**
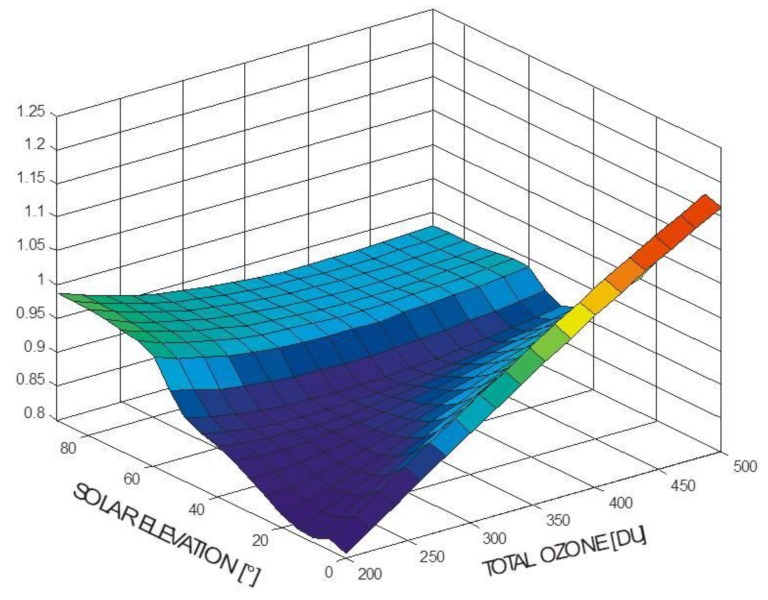
Calibration matrix for a typical broadband UV detector for measuring erythemally weighted solar irradiance showing the relative variation of the calibration factor in dependence on solar elevation and total ozone.

In most cases, monitoring of erythemally weighted irradiance is done by setting up networks of broadband detectors [22,23]. These detectors have a spectral sensitivity which is close to the action spectrum of erythema. Therefore, they can measure signals close to erythemally weighted irradiance immediately with high temporal resolution. However, because of the mismatch (albeit usually small) between the sensitivity of the detector and the erythema action spectrum, combined with the change of the solar spectrum with both solar elevation and ozone content, there is not a simple calibration factor adequate for conversion of the raw signal of the broadband detector to the UVI. A calibration matrix depending on solar elevation and on ozone content has to be applied to each individual reading (Figure 2). Otherwise, errors of up to 20% or more will result [24]. Furthermore, practical experience with this type of detector over long periods has shown that the calibration may change with time in an irregular way. Therefore, it is suggested that calibrations should be performed at least every two years to avoid uncertainties of 20% or more. Hence, measurements with broadband detectors are relatively simple but the calibration needs special care and the data evaluation is also more complicated than for measurements of irradiance at wavelengths longer than UV-B.

In a few networks (e.g., in Norway, Greece) multi-channel filter instruments are used [25]. They measure the irradiance simultaneously at several narrow band wavelengths in the UV-B and UV-A ranges (GUV from Biospherical Instruments, San Diego, CA, USA; NILU-UV from Norwegian Institute for Air Research, Kjeller, Norway; or UV-MFRSR from Yankee Env. Sys. Inc., Turnters Falls, MA, USA). These instruments use stacks of interference and blocking filters in a hermetically sealed and temperature stabilized housing. Their operation is similar to broadband detectors (or a series of such detectors) but they provide some crude spectral information. The spectral response functions of the filters are combined with radiative transfer modeling to generate look-up tables in dependence on solar elevation. These are used with linear combinations of the detector signals to derive the UVI [26]. Further data products are total ozone amount utilizing a pair of channels in the UV-B and UV-A wavelength range and cloud optical depth in the UV-A. However, calibration requirements and long-term stability are as challenging as for broadband detectors (e.g., Reference [27]).

In recent years, diode array instruments have also been used for measurements of erythemally weighted irradiance. This type of instrument typically uses a charge-coupled device (CCD) detector, which is illuminated by the spectrum produced from a monochromator. Therefore, it can measure the spectral intensity at all wavelengths simultaneously within seconds, thus a high measurement frequency of full spectra is possible. Furthermore, it allows for weighting with any action spectrum in post-processing. However, because of the principle of operation, diode array instruments are single monochromators and so the consideration of stray light in the UV-B wavelength range needs special care and increases the uncertainty in the final product. Therefore, at the moment, diode array instruments are generally not well suited to measurements of the UVI [28], but further developments in the future may reduce the drawbacks and take advantage of the rapid scan time.

For the public health context, it is important that information about the relevant level of erythemally weighted irradiance is brought to the attention of the people as fast as possible, because any information about yesterday’s levels is only of ‘historic’ interest. In order to effectively influence exposure and behavior while people are undertaking outdoor activity, the information has to be provided in near real time. Of course, it is also desirable to have a forecast of expected irradiance and UVI levels, but this is—especially under conditions with changing cloudiness—a difficult and uncertain task. Nowadays, the best way for fast data publication is the Internet, which can be accessed from mobile phones, so that relevant information about the UVI locally and at the time can easily reach the interested public.

To provide a reasonably ′personalized′ (i.e., location specific) product, a quite dense network of stations will be necessary, especially in areas with significant variations of topography so that the variability of the erythemally weighted irradiance due to differences in altitude will be observed. Further criteria for selection of measurement sites are large concentrations of population or areas where many people stay for recreation. In addition, areas with different typical weather patterns (e.g., on different sides of mountain ridges) are relevant additions to the measurement sites.

## 4. Monitoring Examples

Worldwide a large number of UV networks for informing the public via the Internet exist. These networks are operated by public or private organizations on a national level (e.g., Reference [29,30,31]). The UVI is always used as the presented quantity, but the frequency of the update of the UVI from the measurement stations varies from minutes to hours. In a recent survey, the monitoring sites in Europe were investigated and a total number of 160 stations in 25 European countries described in terms of instrumentation, quality assurance/quality control (QA/QC), and publication of data to the Internet [32]. Most stations present the results of the measurements as graphs, showing the diurnal variation. In some cases, the actual UVI is given only as a number and sometimes only the maximum value of the UVI around noon is presented. In most web-presentations the value of the UVI is also combined with its respective color, as suggested by WHO [4].

**Figure 3:**
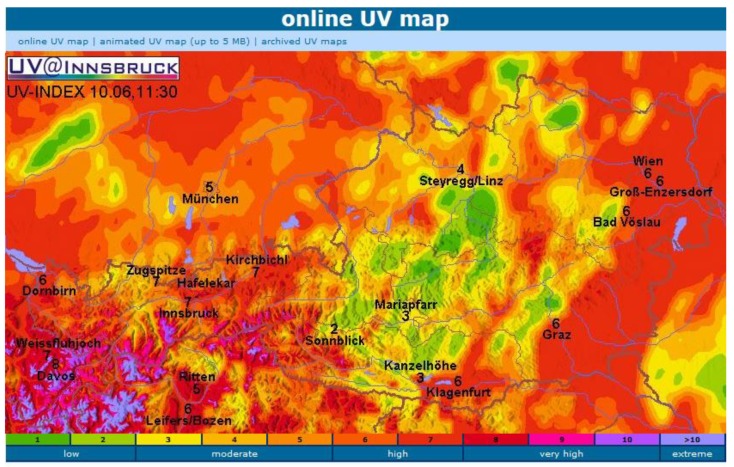
Map of the regional distribution of the UVI over Austria on 10 June 2018 at 11:30 local time. The locations of the measurement stations are marked with their measured UVI. In the south-east of the map the weather is clear, so that the UVI is dominated by the local topography. In the north-east, middle, and south-west, thick clouds reduce the UVI significantly.

To illustrate the complexities of providing a detailed public health UVI product we use the example of UV monitoring in Austria where the complex terrain is an additional challenge. There are currently 13 stations in operation and most of them have been running for 20 years. The measurement sites, at altitudes between 150 and 3100 m above sea level, provide good cover of the whole range of variation of altitudes in Austria. The sites are located in urban and rural areas and furthermore they were selected with respect to spatial representativeness [33]. The measurement sites of the Austrian UV monitoring network are equipped with broadband detectors. Every year, every detector is brought to the reference station in Kirchbichl for a few days, where in the laboratory the relative spectral sensitivity is determined [34]. Then, the absolute sensitivity is determined by comparison with a high-resolution double monochromator spectroradiometer (DTM300 from Bentham Instruments Limited, Reading, UK) operating simultaneously side by side with the test radiometer. The absolute calibration of the spectroradiometer is traceable to Physikalisch Technische Bundesanstalt Braunschweig, Germany, via standard lamps. Furthermore, the spectroradiometer is checked about every five years by comparing it to the travelling reference spectroradiometer, operated by the World Meteorological Organization (WMO) UV reference laboratory Physikalisch-Meteorologisches Observatorium Davos, Switzerland [35]. The difference between the Austrian spectroradiometer and the reference instrument was always less than 3%. The absolute uncertainty of the data of the UVI of the network is estimated to be less than 8% (coverage factor k = 2) because of the absolute calibration chain. The relatively high frequency of recalibration of the broadband detectors was found to be necessary, because some detectors showed changes of 10 to 20% from one year to the next, whereas other instruments were stable within a few percent. The raw data of each detector are transmitted every 10 min to the central server, where the calibration matrix is applied and the UVI is determined. For each station, the UVI is shown in near real time on the web page www.uv.index.at. In addition, a regional generalization of the UVI is presented [36]. The resulting map (Figure 3) is based on the measurements at the time at the available locations and on coincidental information about the regional distribution and density of cloudiness as observed by the weather satellite Meteosat Second Generation (MSG). For the determination of the UVI at each pixel of the map, a clear sky model calculation is first used, taking into account the local ozone value (forecast by National Oceanic and Atmospheric Organisation Global Forecast System (NOAAGFS)), local albedo (snow covered or snow free), climatological aerosols, and the altitude. Then, this value is attenuated by a cloud modification factor. This factor is calculated as the ratio between the irradiance determined with the actual situation of cloud coverage and the irradiance under cloudless conditions. Therefore, it is equal to 1 for cloudless conditions and <1 otherwise. It is derived from the satellite information about cloudiness (optical depth of clouds) and adapted for the UV wavelength range [37]. A final correction is applied, which is based on the comparison of the calculated value with the actual measurement at each measurement site and on a smooth regional interpolation of the difference. Every 15 min, a new image from the satellite is received and an updated map is determined, representing each pixel in the region displayed by its standardized UVI color code.

## 5. Discussion and Conclusions

Overall, good information about the actual level of erythemally weighted irradiance can be found for many locations. However, as already mentioned, for assessing the potential for harm via the individual dose of exposure, the product of irradiance and exposure time is essential. In order to facilitate the information to the public about the dose of solar irradiance, a meaningful suggestion is to introduce the ′UV Index hour′ as a simple quantity, which can be understood intuitively [38]. From the definition of the UVI, it can be derived that 1 UVIh corresponds to 90 Jm^−2^ of erythemally weighted exposure. This dose can then be related easily to the individual minimal erythema dose (MED), which ranges from about 100 Jm^−2^ to 1000 Jm^−2^ for un-tanned skin, depending on the skin type. In addition, a minimum sun protection factor (SPF) can then be estimated in a straightforward manner (where for example SPF 20 reduces the incident UVI to 1/20 of the unprotected value, assuming it is applied at the correct concentration). However, when making such estimations it has to be considered that the basic quantity, the UVI, is determined for a flat horizontal surface. Therefore, the relevance of this information for the exposure of parts of the human body is limited, as the orientation of body parts relative to the sun is very variable. Investigations of the relation between the horizontal dose and personal exposure of different parts of the body show large variations (e.g., Reference [39,40,41,42]), and furthermore remain strongly dependent on clothing and on behavior.

Besides erythemally weighted UV irradiance, it is also important to mention that UV-A irradiance plays a significant role for human health. There are clear detrimental effects of exposure to high UV-A doses on human skin other than premature skin aging. UV-A irradiance affects the lipids of cells resulting in depressed cell metabolism. Furthermore, UV-A exposure can generate highly reactive chemical intermediates, which can damage DNA and contribute to development of skin cancer. Therefore, in parallel to avoiding overexposure to erythemally weighted UV irradiance, it is also important to avoid overexposure to UV-A radiation (e.g., sun screens have to have also an UV-A protection factor).

The large number of measurements of the UVI disseminated worldwide allows interested people to attain information about the expected UV levels at destinations of travel at the time, e.g., for holidays. This provides an important public health service as for many people the annual exposure to erythemally weighted irradiance originates in large part from vacation time. The efforts required to provide high-quality data for this purpose, as illustrated here, should not be overlooked.
